# From control to elimination: a spatial-temporal analysis of malaria along the China-Myanmar border

**DOI:** 10.1186/s40249-020-00777-1

**Published:** 2020-11-19

**Authors:** Fang Huang, Li Zhang, Jing-Bo Xue, Hong-Ning Zhou, Aung Thi, Jun Zhang, Shui-Sen Zhou, Zhi-Gui Xia, Xiao-Nong Zhou

**Affiliations:** 1 National Institute of Parasitic Diseases, Chinese Center for Disease Control and Prevention, Chinese Center for Tropical Diseases Research, WHO Collaborating Center for Tropical Diseases, National Centre for International Research on Tropical Diseases, Ministry of Science and Technology, Key Laboratory of Parasite and Vector Biology, Ministry of Health, Shanghai, 200025 China; 2 Yunnan Institute of Parasitic Diseases, Puer, 665000 China; 3 Department of Public Health, Ministry of Health and Sports, Nay Pyi Taw, 15011 Myanmar; 4 Health Poverty Action East Asia Programme Office, Kunming, 650000 China

**Keywords:** Malaria, Control, Elimination, China–Myanmar border

## Abstract

**Background:**

Malaria cases have declined significantly along the China-Myanmar border in the past 10 years and this region is going through a process from control to elimination. The aim of this study is to investigate the epidemiology of malaria along the border, will identify challenges in the progress from control to elimination.

**Methods:**

National reported malaria cases from China and Myanmar, along with the data of 18 Chinese border counties and 23 townships in Myanmar were obtained from a web-based diseases information reporting system in China and the national malaria control program of Myanmar, respectively. Epidemiological data was analyzed, including the number of reported cases, annual parasite index and proportion of vivax infection. Spatial mapping of the annual parasite index (API) at county or township level in 2014 and 2018 was performed by ArcGIS. The relationship of malaria endemicity on both sides of the border was evaluated by regression analysis.

**Results:**

The number of reported malaria cases and API declined in the border counties or townships. In 2014, 392 malaria cases were reported from 18 Chinese border counties, including 8.4% indigenous cases and 91.6% imported cases, while the highest API (0.11) was occurred in Yingjiang County. There have been no indigenous cases reported since 2017, but 164 imported cases were reported in 2018 and 97.6% were imported from Myanmar. The average API in 2014 in 23 Myanmar townships was significantly greater than that of 18 Chinese counties (*P* < 0.01). However, the API decreased significantly in Myanmar side from 2014 to 2018 (*P* < 0.01). The number of townships with an API between 0 and 1 increased to 15 in 2018, compared to only five in 2014, while still four townships had API > 10. *Plasmodium vivax* was the predominant species along the border. The number of reported malaria cases and the proportion of vivax infection in the 18 Chinese counties were strongly correlated with those of the 23 Myanmar townships (*P* < 0.05).

**Conclusions:**

Malaria elimination is approaching along the China-Myanmar border. However, in order to achieve the malaria elimination in this region and prevent the re-establishment of malaria in China after elimination, continued political, financial and scientific commitment is required.

## Background

Malaria is a life-threatening disease caused by *Plasmodium* parasites that are transmitted to human through the bites of infected female *Anopheles* mosquitoes. It remains one of the most common infectious diseases in the world, with the number of confirmed cases estimated at 228 million with 405 000 deaths in 2018, compared with 416 000 estimated deaths in 2017 and 585 000 in 2010 [1]. The emergence and spread of artemisinin resistant *Plasmodium falciparum* has become one of the greatest challenges to malaria control and elimination in the Greater Mekong Subregion (GMS) [2–5]. Recent efforts to fight malaria in the GMS have yielded impressive results. According to the latest World Health Organization (WHO) estimates, the reported number of malaria cases fell by 76% between 2010 and 2018, and malaria deaths fell by 95% over the same period [1]. Driven by the artemisinin resistance, WHO has implemented a strategy to eliminate *P. falciparum* from six countries in the GMS by 2025, in response to the threat of an untreatable multi-drug resistant parasite [6]. Representatives from six countries signed the “Ministerial Call for Action to Eliminate Malaria in the Greater Mekong Subregion by 2030” in 2018 [7].

**Fig. 1:**
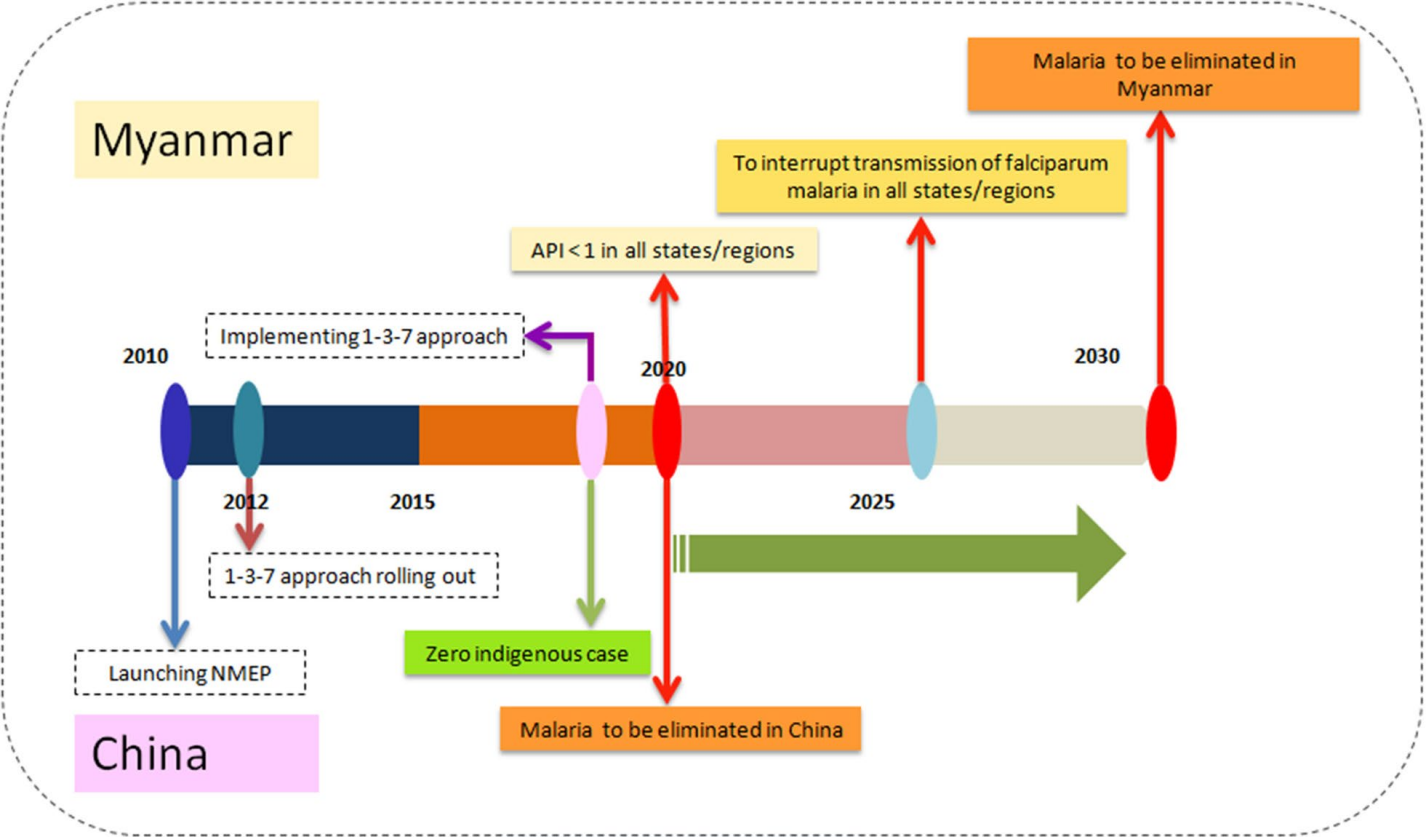
Timeline and milestones of malaria elimination in China and Myanmar. *NMEP* National Malaria Elimination Programme

Historically, malaria was one of the most serious infectious diseases in China [8]. During the past six decades, China has made great contributions towards malaria control [8]. In 2010, the Chinese government launched the National Malaria Elimination Programme (NMEP) 2010–2020 with the goal of eliminating malaria by 2020, sustaining a malaria-free status and prevent re-establishment beyond 2020 (Fig. 1) [9, 10]. Over the following 5 years, malaria cases decreased substantially. In 2017, no indigenous malaria cases were reported for the first time [11]. In 2020, China is close to malaria elimination nationwide. However, Yunnan Province in southern China, which borders Myanmar, Vietnam and Laos, remains the key focus of the NMEP.

Myanmar has the highest malaria burden and is a major source of malaria exportation in the GMS [12, 13]. In the past decade, Myanmar has made significant progress in reducing malaria morbidity and mortality with the financial support for major improvements in access to early diagnosis and appropriate treatment. Malaria morbidity was reduced by 72% in 2016 compared with 2012 and there was a 95% reduction in malaria deaths within the same period [14]. However, Myanmar was still reported to account for the majority of malaria cases and deaths in the GMS [7, 15, 16]. Meanwhile, conflict-affected settings and regions with high population mobility have enhanced the programmatic challenges of moving towards elimination [17–19]. Following the recommendation by the WHO Malaria Policy Advisory Group to eliminate *P. falciparum* malaria in the GMS by 2030 in 2014, which was technically, operationally and financially feasible, in 2016 the National Malaria Control Programme (NMCP) in Myanmar set the goal of eliminating *P. falciparum* malaria by 2025 and eliminating malaria in all states and regions by 2030 (Fig. 1) [20].

A total of 18 counties in Yunnan Province, China and 23 townships in Myanmar share the border of 1997 km. The border areas on both sides are outlying, hard to reach and poverty-stricken inhabited by the minority nationalities [21]. Currently, there are 13 national and provincial frontier ports, 427 passageways and countless shortcuts along the border. The climate, landscape and vectors of malaria transmission on both sides of the border are similar. In the last 10 years, malaria incidence has declined remarkably and the progress was made possible through greater access to effective malaria control tools, particularly artemisinin-based combination therapies (ACTs) for malaria treatment, rapid diagnostic tests (RDTs), and insecticide-treated nets (ITNs). Therefore, we undertook a spatial and temporal analysis to investigate the changing pattern of malaria along this border and identify the key priorities and challenges in the progress from malaria control to elimination.

## Methods

### Study site

**Fig. 2:**
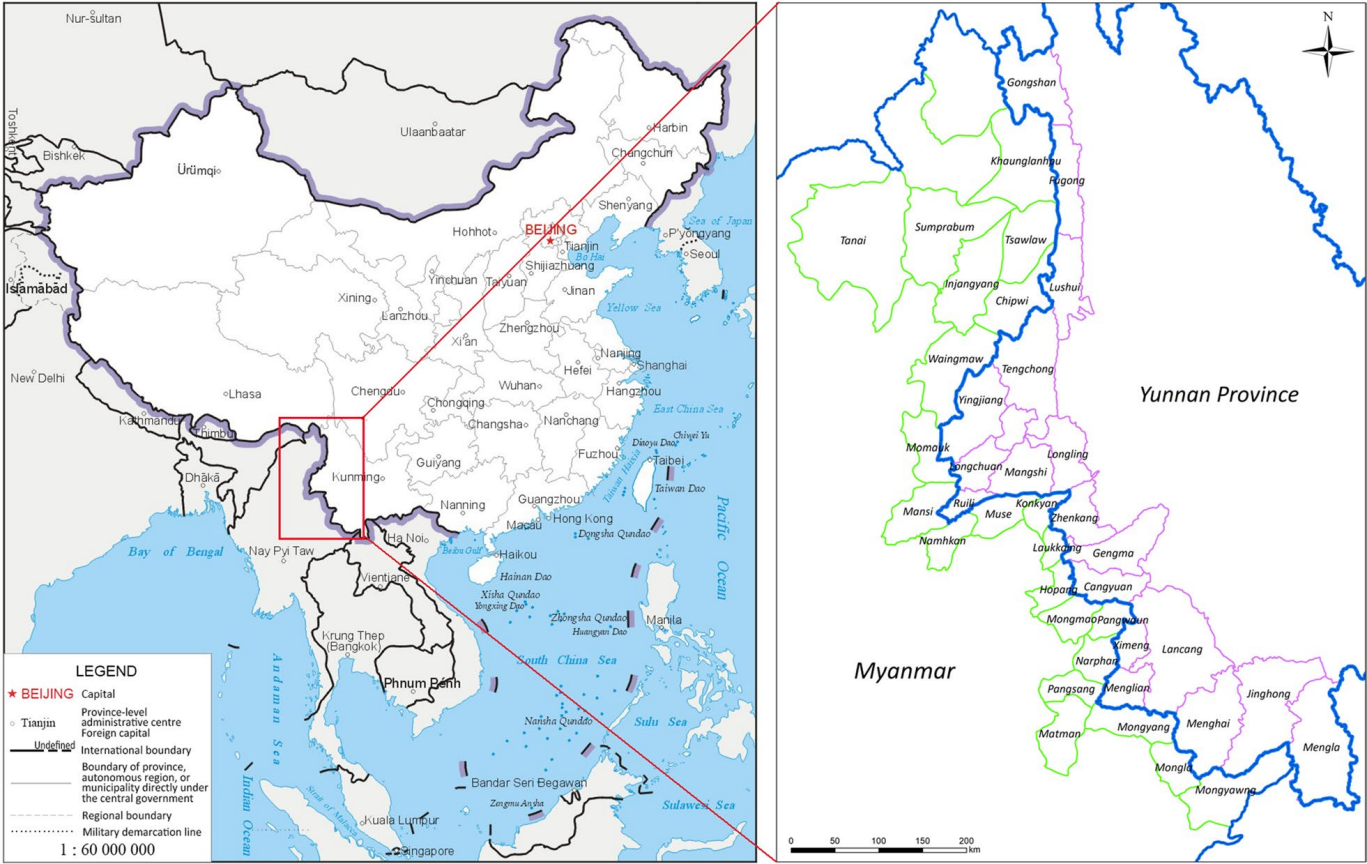
Map of the borders between the China and Myanmar. The China map is from National Geomatics Center of China with the link https://www.ngcc.cn/ngcc/. And the map is correct by checking with that from the web link: https://bzdt.ch.mnr.gov.cn/

The study areas include 18 counties in Yunnan Province, China and 23 townships in Kachin, North Shan and East Shan states of Myanmar (Fig. 2).

Yunnan Province, located in the southern China, spans approximately 394 000 km^2^ with a population of 48.3 million. Yunnan has high mountains bordering Tibet and Sichuan in the west, a hilly plateau in the east bordering Guangxi and Guizhou provinces/autonomous regions, and a tropical zone in the south sharing the border with Vietnam, Laos, and Myanmar. The average daily high temperature is around 24° centigrade and the climate is almost moderate, but also offers a few sultry months with high humidity and high temperatures. In the western Yunnan, it has distinct dry and rainy seasons. A total of 18 counties lie along the border with Myanmar.

Kachin State, also called Jinghpaw Mung is the northernmost state of Myanmar. It is bordered by Tibet and Yunnan in China in the north and east, respectively. The average temperature in Kachin state is 25° centigrade and the average precipitation of rainfall is around 2100 mm per year. The rains are very abundant from June to August. Kachin State covers four districts, Myitkyina, Bamaw, Moehnyin, and Putao and 18 townships. The population is approximately 1.6 million. Ten townships in Kachin share borders with China. Shan State, located in the northeast of Myanmar, is traditionally divided into three sub-states: North Shan, East Shan, and South Shan states. Shan State covers 155 800 km^2^, almost a quarter of the total area of Myanmar. Most of Shan State is a hilly plateau, which together with the higher mountains in the north and south forms Shan Hills system. Eleven townships in North Shan state, and two from East Shan State border China to the north. The total population is around 5.8 million.

### Case data collection

Malaria data from China were collected from the Chinese Infectious Disease Report System (CIDRS), a web-based reporting system for individual case and data management for notifiable infectious diseases from 2005 to 2018. The classification of malaria cases in China was made according to the criteria from the national guidelines, and included epidemiologically indigenous and imported cases as well as laboratory-confirmed cases defined as cases confirmed using any of the diagnostic tests, such as polymerase chain reaction (PCR), rapid diagnosis test (RDT), and microscopy examination and clinically diagnosed cases defined as patients with malaria-like symptoms without detection of parasites through blood examination [22]. The malaria case data from 18 border counties was extracted accordingly for mapping the malaria distribution at the county level.

Malaria data from Myanmar was obtained from the NMCP. The cases included probable malaria cases and confirmed cases, defined as positive by diagnostic testing including microscope examination and/or RDTs. The malaria cases and related epidemiological data from 23 border townships were collected by NMCP along with another five non-government organizations: Human Poverty Action, Myanmar Council of Churches, Myanmar Medical Association, Medical Action Myanmar, and Population Service International.

### Data analysis

Data analysis was processed using Microsoft Excel 2017 and SAS software (SAS Institute Inc, Version 9.2, Cary, NC, USA). Annual parasite index (API) is the definition of the number of confirmed new cases from malaria registered in a specific year, expressed per 1000 individuals under surveillance, for a given country, territory, or geographic area. API = total confirmed cases in a year × 1000/total population. The APIs were transformed to logarithms with a base of 2.5 to attain a normal distribution and homogeneity of variance. APIs of 23 Myanmar townships and 18 Chinese counties in 2014 and APIs of the former and imported malaria cases of the latter in 2018 were mapped by using ArcGIS 10.1 (Environmental Systems Research Institute, Inc, Redlands, CA, USA) to identify the geographical distribution of malaria on both sides. The relationship between the number of reported malaria cases and APIs on both side of the border was analyzed by a linear regression mode. A *P* value < 0.05 was used to evaluate differences with statistical significance.

## Results

### Malaria endemicity in China and Myanmar at country level

**Fig. 3:**
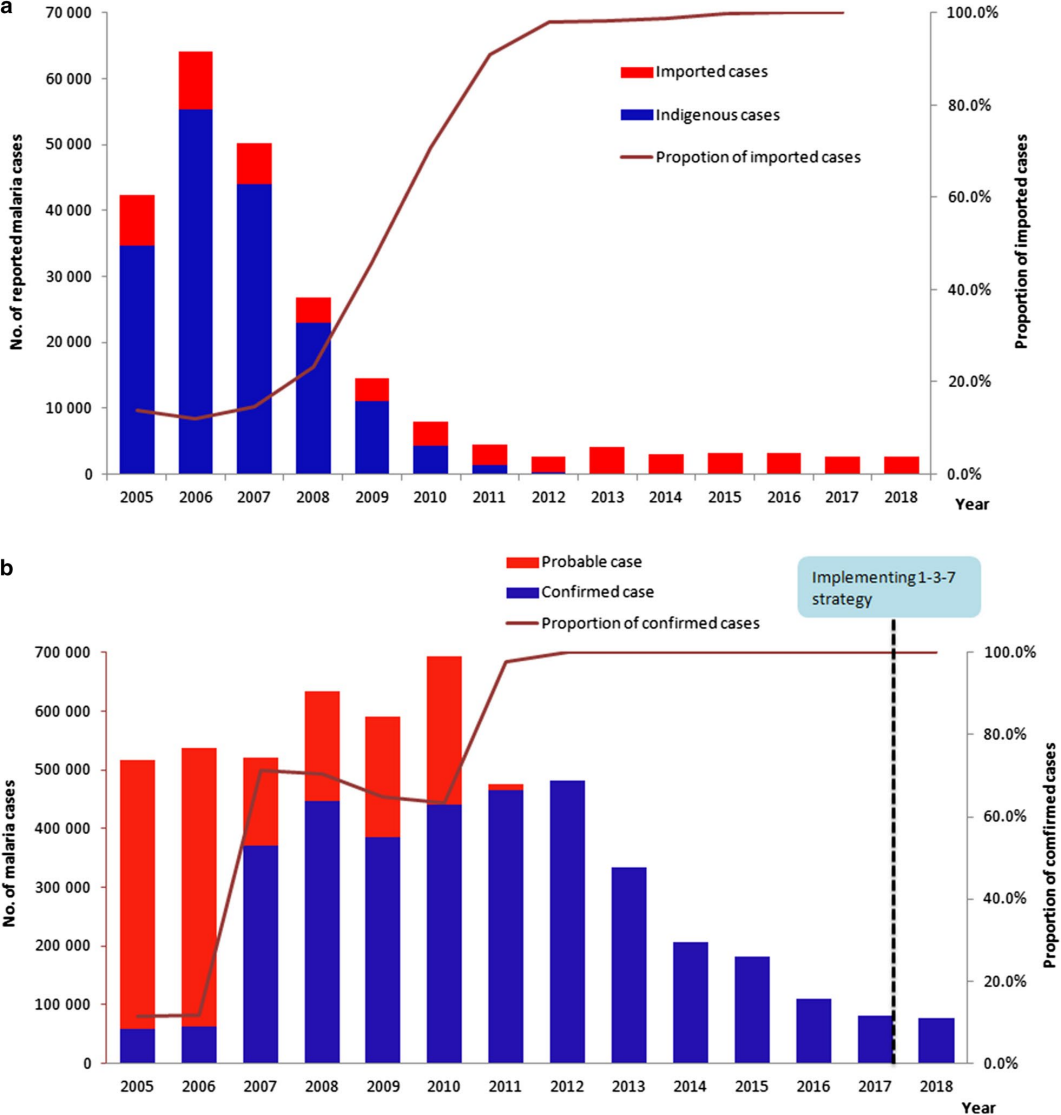
Reported malaria cases in China and Myanmar 2005 to 2018 (**a**, **b**)

Reported malaria cases from China, Myanmar and the border areas were collected to assess the trend of the malaria transmission from 2005 to 2018. The reported cases decreased to a low level with only thousands of cases in 2015–2018 in China compared with the hundreds of thousands of cases before 2010 when NMEP had not been launched. A total of 42 319 malaria cases were reported in the entire country in 2005 and this declined to 2678 cases in 2018 (Fig. 3a). The number of counties with indigenous malaria cases decreased from 1168 in 2005 to one in 2016 and zero in 2017. The proportion of imported cases was 18.3% in 2005 and then increased and was maintained at more than 90% since 2012 and up to almost 100.0% except for a few induced cases from 2017 (Fig. 3a). In 2017, no indigenous cases were reported in the whole country for the first time.

A total of 76 518 malaria cases were reported in Myanmar in 2018 compared with 516 041 in 2005, representing a decline of 85.2% (Fig. 3b). The trend of malaria reported cases presented a single peak pattern. The number of cases steadily ascended from 2005 to 2010 but descended remarkably from 2010 to 2018. The proportion of confirmed malaria cases was only 11.5% (59 405/516 041) and 11.7% (62 813/538 110) in 2005 and 2006, respectively, but has significantly increased since 2007 and reaching to 97.9% (465 294/475 509) in 2011 (Fig. 3b). All the reported malaria cases have been confirmed since 2012.

### Reported malaria cases and API at county or township level

**Fig. 4:**
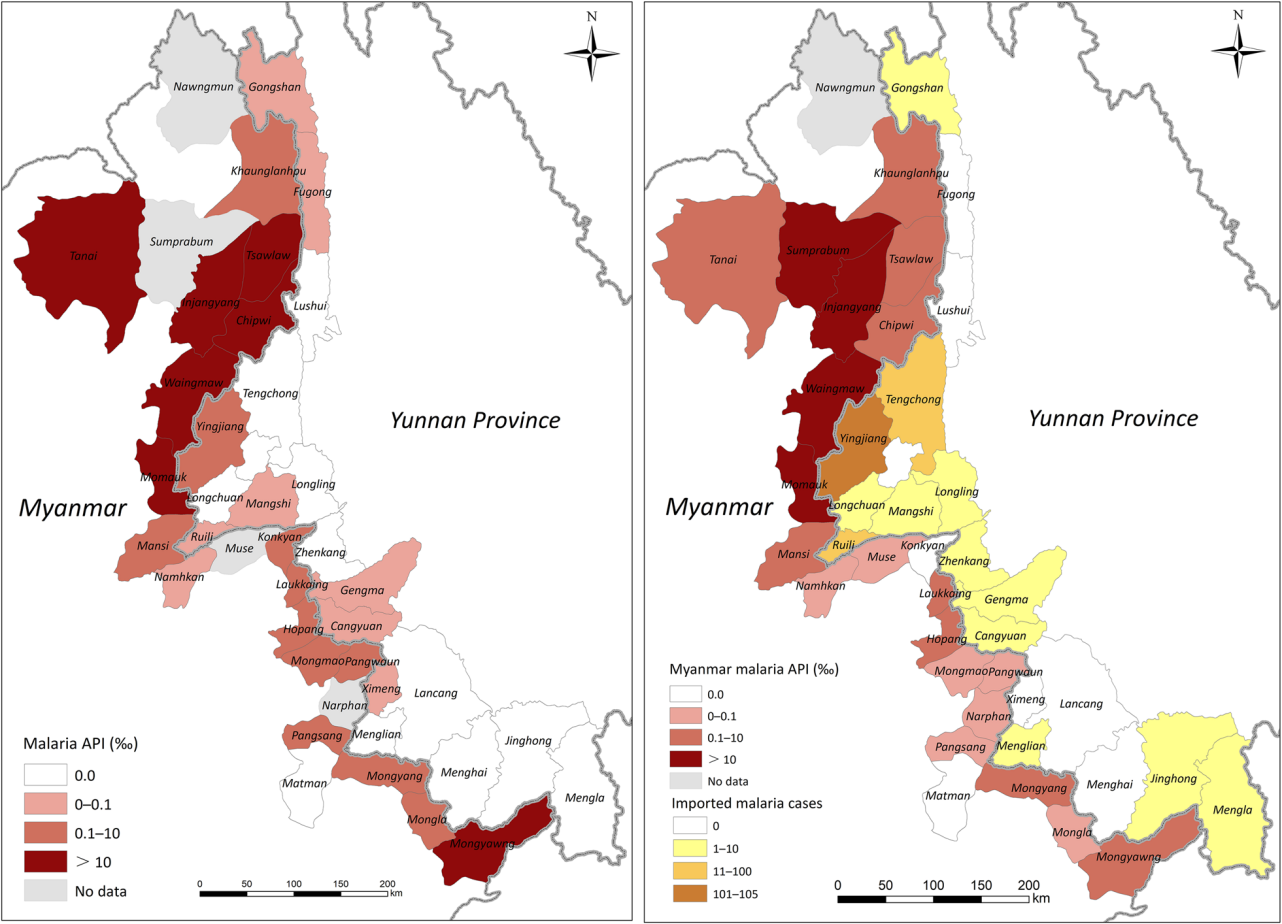
API mapping based on county or township level in 2014 and 2018

Both the number of reported malaria cases and API declined in the border counties or townships on both sides. In 2014, 392 malaria cases were reported from 18 counties in Yunnan Province of China, which included 33 (8.4%) indigenous cases and 359 (91.6%) imported cases. Ten counties reported no indigenous malaria cases and seven counties (accounting for 38.9%, 7/18) displayed an API range of 0–0.1. The highest API (0.11) was occurring in Yingjiang County (Fig. 4a), which was also the last indigenous malaria case as reported in the whole country. Since 2017, no indigenous cases have been reported in China, including these border counties. In 2017, a total of 263 imported malaria cases reported in 18 border counties and 97.0% (255/263) were from Myanmar. The distribution of imported malaria cases in 2018 was mapped (Fig. 4b). There were 164 imported cases reported in 2018 and 97.6% (160/164) were imported from Myanmar (Table 1): 64.0% (105/164) were reported in Yingjiang County, followed by Tengchong (11.0%, 18/164) and Ruili (7.3%, 12/164). In addition, the percentage of *P. falciparum* imported from Myanmar was decline steadily from 2014 to 2018 while the percentage of *P. vivax* imported from Myanmar was no significantly different in the same period (Table 2).

**Table 1:** The infection place of malaria reported cases in 18 Chinese border counties from 2014 to 2018

Year	Imported cases, *n* (%)				Indigenous case, *n* (%)	Total, *n*
	Myanmar	Other counties in GMS	Africa	Others		
2014	351 (89.5)	8 (2.0)	0 (0.0)	0 (0.0)	33 (8.4)	392
2015	447 (93.5)	19 (4.0)	0 (0.0)	1 (0.2)	11 (2.3)	478
2016	299 (95.8)	7 (2.2)	5 (1.6)	0 (0.0)	1 (0.3)	312
2017	255 (97.0)	6 (2.3)	1 (0.4)	1 (0.4)	0 (0.0)^a^	263
2018	160 (97.6)	0 (0.0)	4 (2.4)	0 (0.0)	0 (0.0)^a^	164

*GMS* Greater Mekong Subregion

^a^There was no indigenous malaria cases reported in 2017 and 2018

**Table 2:** The number and percentage of imported malaria cases from Myanmar and other countries according to *Plasmodium* spp.

Year	*P. falciparum* (*Pf*)			*P. vivax* (*Pv*)			*P. malariae*			Mix (*Pf* + *Pv*)		
	Myanmar, *n* (%)	Others countries, *n* (%)	Total	Myanmar, *n* (%)	Others countries, *n* (%)	Total	Myanmar, *n* (%)	Others countries, *n* (%)	Total	Myanmar, *n* (%)	Others countries, *n* (%)	Total
2014	55 (100.0)	0 (0.0)	55	294 (97.4)	8 (2.6)	302	2 (100.0)	0 (0.0)	2	0 (0.0)	0 (0.0)	0
2015	46 (97.9)	1 (2.1)	47	399 (95.5)	19 (4.5)	418	1 (100.0)	0 (0.0)	1	1 (100.0)	0 (0.0)	1
2016	16 (76.2)	5 (23.8)	21	283 (97.6)	7 (2.4)	290	0 (0.0)	0 (0.0)	0	0 (0.0)	0 (0.0)	0
2017	9 (90.0)	1 (10.0)	10	246 (97.2)	7 (2.8)	253	0 (0.0)	0 (0.0)	0	0 (0.0)	0 (0.0)	0
2018	6 (66. 7)	3 (33.3)	9	150 (99.3)	1 (0.7)	151	1 (100.0)	0 (0.0)	1	3 (100.0)	0 (0.0)	3

The average API in 2014 in 23 townships in Myanmar was significantly greater than that of the 18 counties in China (*P* < 0.01). In 2014, only one township Matman in North Shan State, no malaria cases reported. There were five and six townships with API of 0–1 and 1–10, respectively. Seven townships showed API of more than 10 and six out of them were located in Kachin State (Fig. 4). The highest API was 55.2 reported from Injangyang township. In 2018, Matman and Konkyan reported no malaria cases. The API decreased significantly in these 23 townships in Myanmar from 2014 to 2018 (*P* < 0.01). The number of townships with APIs between 0–1 increased to 15 in 2018, compared to only five in 2014 (Fig. 4). However, there were still four townships with APIs of 10–50 and two townships with APIs of 1–10. The highest API was 19.6, in Sumprabum township in northern Kachin State.

### Proportion of *P. vivax*

**Fig. 5:**
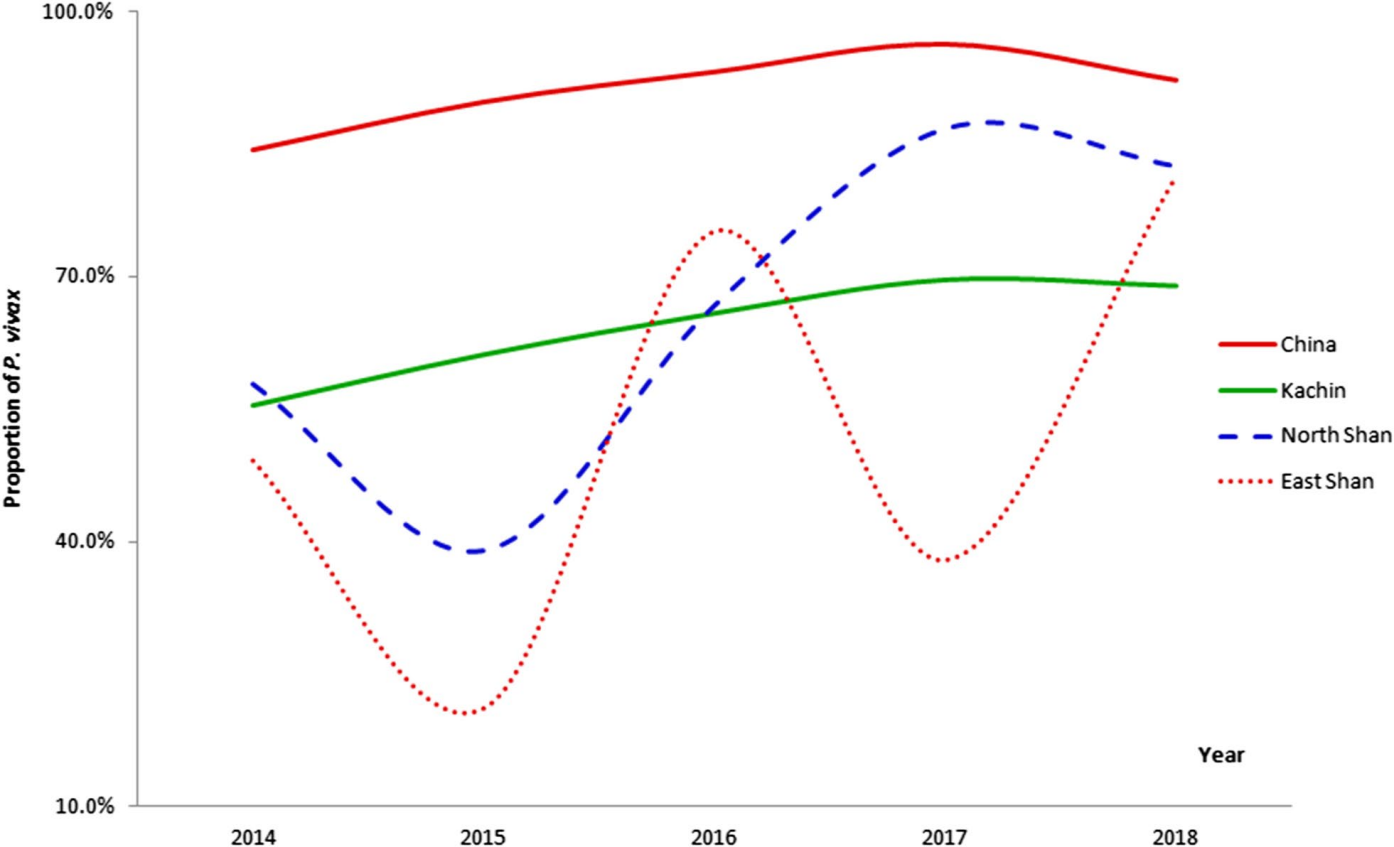
Proportion of *Plasmodium vivax* malaria infection in the 18 counties and 23 townships along the China–Myanmar border

*P. vivax* was the predominant malaria parasite along the China-Myanmar border. In 2014–2018, the average proportion of *P. vivax* infection in the 18 Chinese counties and the 23 Myanmar townships was 91.1% (range: 84.3–96.3%) and 61.2% (range: 37.9–86.8%), respectively (Fig. 5). *P. vivax* infection was more common in China than in Myanmar. The proportion of *P. vivax* cases in North Shan was highest (66.5%), followed by Kachin State and East Shan State. The trend of the proportion of *P. vivax* cases showed a slight increase in the 18 border counties in China, while ascending more on Myanmar side. Interestingly, the proportion of vivax infection was much lower in East Shan in 2017 and increased sharply from 2017 to 2018 while others states showed a small decrease in 2017–2018.

### Seasonality and annual trend of malaria in Chinese border counties

**Fig. 6:**
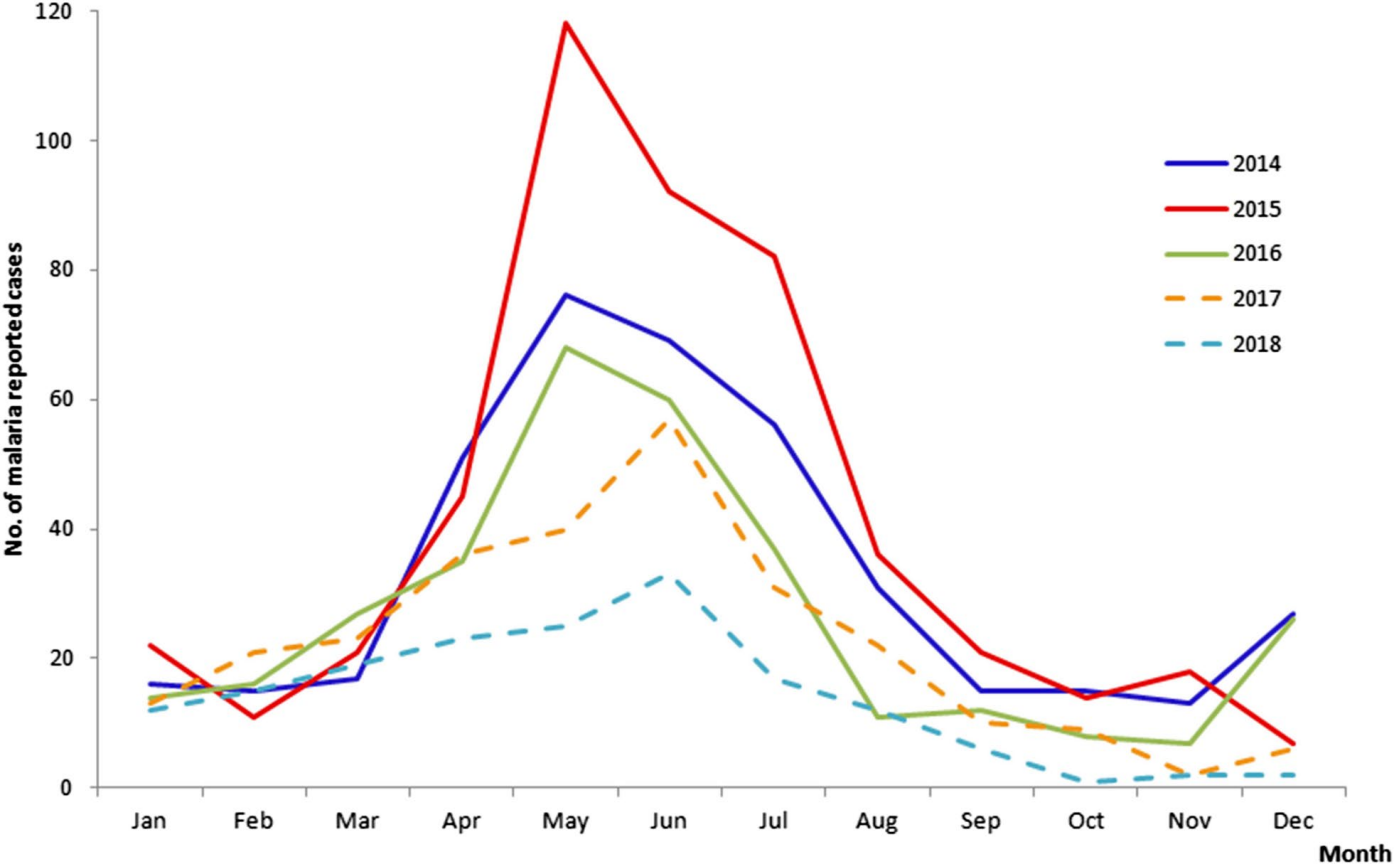
Monthly reported malaria cases in the 18 border counties in China

Reported malaria cases in the 18 border counties in China displayed well-defined seasonality in 2014–2018, with one peak from May to July and a slight peak from December to the following January (Fig. 6). This malaria transmission coincided with the environment and weather which was strongly correlated with the abundance of *Anopheles*. There are only two seasons in this border area, the rainy season from May to September and the dry season from October to the following April. The trend of total malaria cases in these 18 counties showed a steady decline except in 2015. There was only imported cases, with no indigenous malaria cases reported since 2017.

### The relationship of malaria endemicity between two sides of the border

**Fig. 7:**
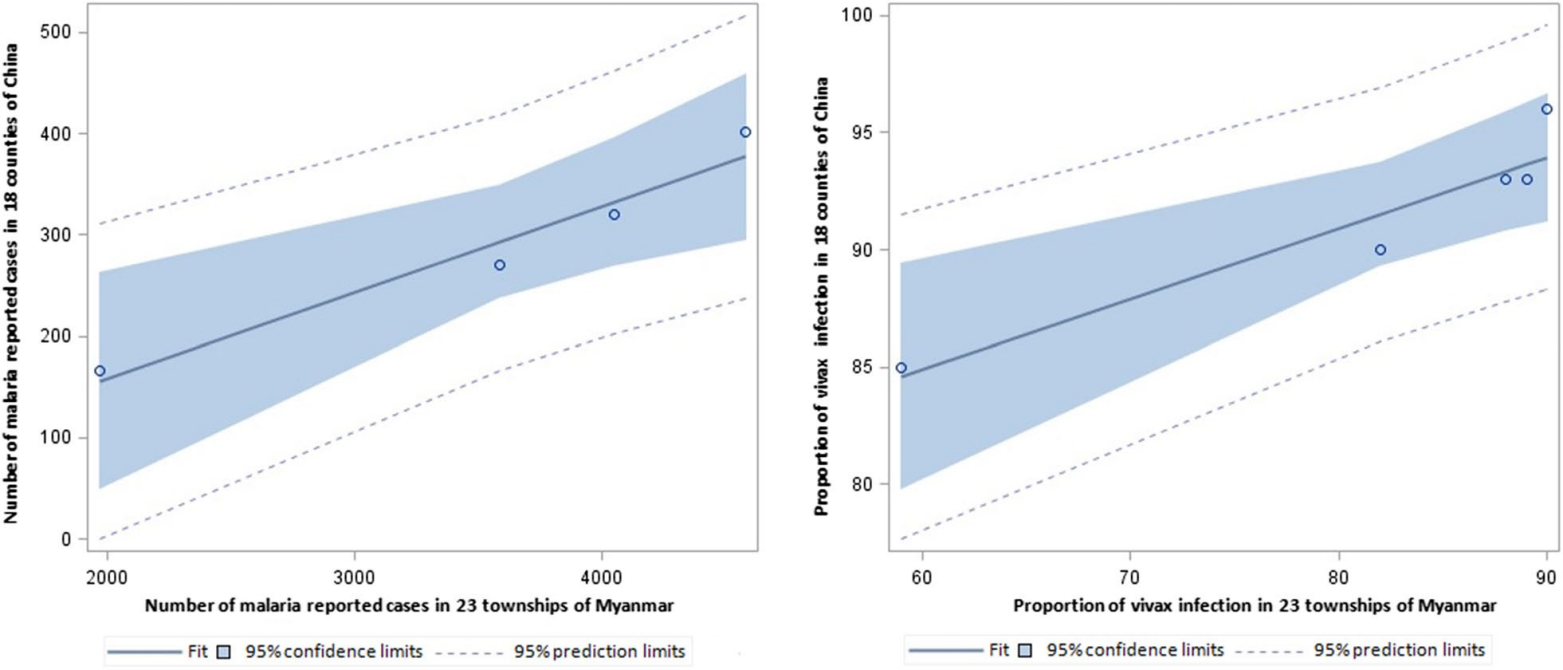
The relationship of the number of reported malaria cases and proportion of vivax malaria infection between both sides of the border

The reported malaria cases and API on both sides decreased dramatically from 2014 to 2018 (Fig. 4). The proportion of vivax infection showed a significant correlation between both two sides of the border (R^2^ = 0.895, 95% *CI:* 51.0471–82.4077). Similar results were obtained for the proportion of *P. falciparum* and mixed infections (R^2^ = 0.9559; 95% *CI:* − 1.6672 to 5.2537) (Fig. 7). The number of reported malaria cases was correlated between the two side, with R^2^ of 0.9522 (95% *CI:* − 223.9488 to 201.2637) after the data for 2015 was removed (*P* = 0.0040). The API data had to be exponentially converted with a base of 2.5 to attain a normal distribution and homogeneity of variance. Interestingly, there was no significantly relationship of the APIs between the two sides of the border (*P* > 0.05).

## Discussion

Malaria control has achieved great progress in China in the past decades, which were caused of effective control of the malaria epidemic and ensuring the health of the population along with the promotion of social and economic development [23]. More intensive work on surveillance and response in malaria endemic areas was seen after the NMEP was launched in 2010 [9, 24, 25]. With a strengthening surveillance and response system, the implementation of surveillance and response was standardized as a “1-3-7” surveillance approach, which means case reporting within 1 day, case investigation within 3 days and focus investigation and action within 7 days [26]. In our study, the total number of reported malaria cases in China fell 94.1% from 2005 to 2018 (Fig. 2).The majority of the counties with indigenous malaria cases reported were located along the China–Myanmar border [27]. There have been no malaria indigenous malaria cases reported in China since 2017 [11] and China is approaching malaria elimination nationwide in 2020.

Myanmar is reported to account for the vast majority of malaria cases and deaths in the GMS [13]. The proportion of confirmed cases in Myanmar was much lower prior to 2012 (Fig. 3b), which may have been caused by limited access to early malaria diagnosis and appropriate treatment in the community. According to the National Plan for Malaria Elimination in Myanmar 2016–2020, the key interventions including early and effective malaria case management, universal coverage of high-risk populations with appropriate malaria prevention measures and case-based surveillance have been implemented for elimination and prevention of re-establishment in Myanmar. The significant progress has been achieved in reducing malaria morbidity and mortality, with the reduction of malaria cases by 72% from 2012 to 2018, with a similar trend found in another study [28]. These achievements reflected a substantial improvement in case diagnosis and treatment and vector control, particularly at the periphery and among populations at risk of malaria, and increasing financial support from the Global Fund and other donors as well [29, 30]. In response to the threaten by the emergence and spread of *P. falciparum* resistant to artemisinin, the world’s first line antimalarial [31] in Cambodia, Thailand, Vietnam, Laos and Myanmar [4, 32, 33], WHO set its sights on malaria elimination in the GMS in order to contain this threat [6]. The National Plan for Malaria Elimination in Myanmar 2016–2030 has been developed with the goal of decreasing the API to < 1 in all states/regions by 2020, interrupting transmission of falciparum malaria in all states/regions by 2025 and eliminating malaria nationwide by 2030 [34, 35].

Although great gains have been made in reducing the overall cases of malaria, achieving an impact from elimination and control efforts proves more difficult in areas near international borders [36, 37]. The specific environmental (including physical, social and geopolitical), anthropological, administrative and geographic characteristics of border areas have a unique impact on the epidemiology of malaria. Cross-border malaria is difficult to manage because of political, economic and geographic constraints [38]. Border malaria is a major obstacle to achieving malaria elimination in the GMS [39]. China and Myanmar share a border of around 2000 km, which includes 18 counties in Yunnan Province of China and 13 townships from Kachin State, eight from Northern Shan State and two from Eastern Shan State in Myanmar [40]. The border counties on Yunnan side had the highest number of reported malaria cases in China [27] and the border townships in Myanmar had a relatively high transmission of malaria as well [41], resulting from lower access to health services, difficulties in deploying the prevention program to hard-to-reach communities, often in difficult terrain, and constant movement of people across porous national boundaries. The border counties in Yunnan Province are the key focus for malaria elimination in China and the 23 townships in Myanmar are one of the most difficult regions to be reached and covered by the NPME. This study showed that both reported malaria cases and API declined in the border counties or townships on both sides, but a few townships in Kachin State still had higher API, such as Injangyang, Momauk and Sumprabum. These townships should be the key focus for malaria elimination along this border.

When compared with *P. falciparum*, *P. vivax* is geographically the most widespread cause of human malaria with over 2.5 billion people living at risk of infection [42, 43]. Vivax malaria has a high prevalence in Southeast Asia, and in Central and South America. As reported by WHO, 53% of the *P. vivax* infection was in the WHO South-East Asia Region [1]. This study found that *P. vivax* was the predominant malaria parasite along the China–Myanmar border and the proportion of* P. vivax* infection increased from 61.6% in 2014 to 81.2% in 2018 with a very small decrease from 2017 to 2018. This may be caused by the increasing imported cases with *P. falciparum* from Africa in Yunnan Province of China and multidrug resistance emerging of *P. falciparum* in GMS including Myanmar borders. Interestingly, the trend of proportion of *P. vivax* infection in East Shan showed the shape of "W". One reason may be some *P. vivax* cases were detected and recognized in remote area, which could be inferred from the small number of reported malaria cases in East Shan, i.e. Mengla only reported less than 10 malaria cases in 2015–2018. In addition, *P. vivax* was more common on Chinese side than on the Myanmar side. The epidemiology of vivax malaria in this region is highly complex and *P. vivax* has become a major challenge for malaria elimination in the GMS [13, 44].

The number of reported malaria cases and the proportion of vivax infection in the 18 Chinese counties was strongly correlated with those in the 23 townships of Myanmar (*P* < 0.05). Interestingly, we did not find a correlation of API between the two sides. API is defined as the number of confirmed new cases expressed per 1000 individuals under surveillance in a specific year, and it usually refers to areas of high and moderate malaria transmission risk [45, 46]. The study data was obtained from the China-Myanmar border, which showed low to moderate transmission of malaria with a large mobile population and local population. This may lead to the bias when API is used as an indicator for regression analysis.

In addition, the similar climate and environment along the border area played one major role in malaria transmission, which were mostly through its effects on both the mosquito vector and the development of the malaria parasite inside the mosquito vector [47]. The diversity of malaria vectors in Yunnan Province was the highest in China. A total of 53 *Anopheles* species have been found, accounting for 88.3% of all *Anopheles* species reported in China. At least five *Anopheles* species or complexes were recorded as the predominant malaria vector in Yunnan Province, i.e. *An. minimus*, *An. sinensis*, *An. kunming*, *An. anthropophagus*, and *An. dirus* [38, 48]. *An. minimus* is the primary malaria vector along the China–Myanmar border.

The density of population and economic development in 18 Chinese border counties were higher than the border townships in Myanmar, which may have an indirect effect on the reduction of malaria [40]. Mobile population is also one of the risk factors for malaria control and elimination. A high proportion of mobile population was associated with greater malaria vulnerability in the China-Myanmar border region and the population-specific strategies and measures would be useful to decrease the risk of malaria re-establishment in China [38, 49]. Since 2014, a cross-border malaria prevention and control cooperation mechanism has been established between China and Myanmar to accelerate the control and elimination of malaria in this border region, and the strategic plan was drafted with "one zone one strategy" to promote the joint actions [7]. Efforts are underway to strengthen surveillance and to enhance reporting from the private sector and nongovernmental organizations (where relevant), with case-based surveillance and a response accelerating towards elimination. Based on the latest malaria epidemiology on the border, it is necessary to further promote the updating and implementation of this cooperation strategy and its action plan, highlighting the areas with high API in Myanmar side and high risk of malaria re-establishment in China side for achieving and maintaining the elimination in both the countries. Another point should be considered is that COVID-19 pandemic is affecting the global malaria control and elimination. WHO have published documents to provide guidance to ensure the maintenance essential malaria services at different level while working to control COVID-19 [50, 51].

One limitation of this study is that malaria data was analyzed in a large scale, which was based on the county or township level. However, malaria cases were more scattered in the villages or communities in the pre-elimination or elimination stage. The further spatial-temporal analysis of malaria in small scale at village or community level will be more accurate.

## Conclusions

There has been a dramatic declined in malaria incidence along the China-Myanmar border and malaria elimination is approaching along the China-Myanmar border. However, in order to achieve the malaria elimination in this region and prevent the re-establishment of malaria in China after elimination, continued political, financial and scientific commitment and joint actions are still required.

## Data Availability

The datasets used and/or analysed during the current study are available from the corresponding author on reasonable request.

## Abbreviations

ACTs: Artemisinin-based combination therapies
API: Annual parasite index
GMS: Greater Mekong Subregion
ITNs: Insecticide-treated nets
NMCP: National Malaria Control Programme
NMEP: National Malaria Elimination Programme
PCR: Polymerase chain reaction
RDT: Rapid diagnostic test
WHO: World Health Organization
